# Smartphone-enabled otoscopy: method evaluation in clinical practice

**DOI:** 10.1016/j.bjorl.2021.08.012

**Published:** 2021-11-15

**Authors:** Fernanda Dal Bem Kravchychyn, Ana Taíse de Oliveira Meurer, Maria Helena Salgado Delamain Pupo Nogueira, Fernando Oto Balieiro, Fernando de Andrade Balsalobre, Iulo Sérgio Barauna Filho, Aldo Eden Cassol Stamm

**Affiliations:** Hospital Edmundo Vasconcelos, São Paulo, SP, Brazil

**Keywords:** Otoscopy, Otology, Smartphone, Otoendoscopy, Telemedicine

## Abstract

**Objective:**

To assess the diagnostic agreement between smartphone-enabled otoscopy and rigid otoendoscopy in tympanic membrane and middle ear diseases.

**Methods:**

A cross-sectional study was carried out to analyze otoscopies in patients seen at a general otorhinolaryngology (ORL) outpatient clinic, from June to December 2019. Eighty-three images of patients obtained from otoscopies performed through a smartphone device and a rigid endoscope were included, recorded, and stored for further analysis. The images were first analyzed by an experienced otologist, who assigned his diagnostic impression (defined as the gold standard) on each of the images. After this analysis, the images were displayed to a group of secondary raters (an experienced otorhinolaryngologist, a second-year resident in ORL, and a general practitioner). A questionnaire was applied related to each image.

**Results:**

There was high agreement between the smartphone device and the otoendoscopy images for all professionals, with a Kappa coefficient of 0.97 (*p* < 0.001). The smartphone device showed a diagnostic sensitivity of 81.1% and a specificity of 71.1%. As for the otoendoscopy, it showed a sensitivity of 84.7% and a specificity of 72.4%. The image classification as “2 = Good” was the most frequent one, with 34.9% for otoendoscopy and 31.6% for the smartphone device.

**Conclusion:**

There was a high diagnostic agreement between smartphone device-guided otoscopy and the rigid otoendoscopy, demonstrating the feasibility of using this device in clinical practice.

Level of Evidence: 3

## Highlights

• The images allowed a reliable diagnosis of ear pathologies.
• There was a high diagnostic agreement between the two evaluated methods.
• The feasibility of using the device in clinical practice was demonstrated.

## Introduction

Otoscopy is an essential resource in the evaluation of the tympanic membrane (TM) and is crucial in otorhinolaryngological propaedeutics.^1^ Toynbee, a 19^th^-century British otologist, was the first to devise a speculum with an oval lumen that would better fit the ear canal, allowing better visualization of the membrane. This was the initial milestone that resulted in the further development of several semiological resources, such as otoendoscopy.^2^, ^3^

Otoendoscopy has introduced a new dimension in the observation of TM, since the rigid endoscopy combined with lighting by cold light sources allows superior image acquisition quality when compared to the conventional otoscopy.^4^, ^5^, ^6^ Moreover, it allows image capture when coupled to photographic or video recording devices.

However, it is still an inaccessible resource on a large scale, especially in the outpatient and hospital setting, due to the high cost associated with endoscopes and high-resolution image capture systems.^7^, ^8^ The ubiquity associated with the advent of smartphones allowed the development of portable, high-tech and affordable otoendoscopy devices.^9^ These smartphone gadgets allow them to become a portable otoscopic system, offering high resolution image and video capture, as they use features such as focus and lighting from the smartphone itself.^10^, ^11^

The practical features of this system provides real-time images that allow instant feedback to the patient during the consultation, in addition to facilitating the understanding and care in relation to the disease. The immediate availability of images allows remote access at any distance, allowing several advantages, such as the development of medical education and telemedicine and, in a current perspective, being the substrate for the development of image mapping applications and artificial intelligence systems aiming at diagnostic accuracy.^12^, ^13^, ^14^

The aim of this study is to evaluate the diagnostic agreement between smartphone-enabled otoscopy and rigid otoendoscopy in tympanic membrane and middle ear diseases.

## Methods

A cross-sectional study was carried out to analyze otoscopies in patients seen at a general otorhinolaryngology clinic, from June to December 2019. Adult male and female patients, over 18 years of age, with and without the presence of pathologies observed during conventional otoscopic examination were included. After this initial assessment, the patient was asked for permission to capture images through the otoscopy devices used in the study (rigid otoscopy and smartphone device otoscopy), both connected to a recording system. Once accepted by the patients, the Free and Informed Consent form (FICF) was applied. Patients who had cerumen that completely occluded the external auditory meatus were excluded from the study, due to the possibility of resulting in compromised quality images or even dubious diagnosis, due to possible trauma during the removal procedure.

**Figure 1:**
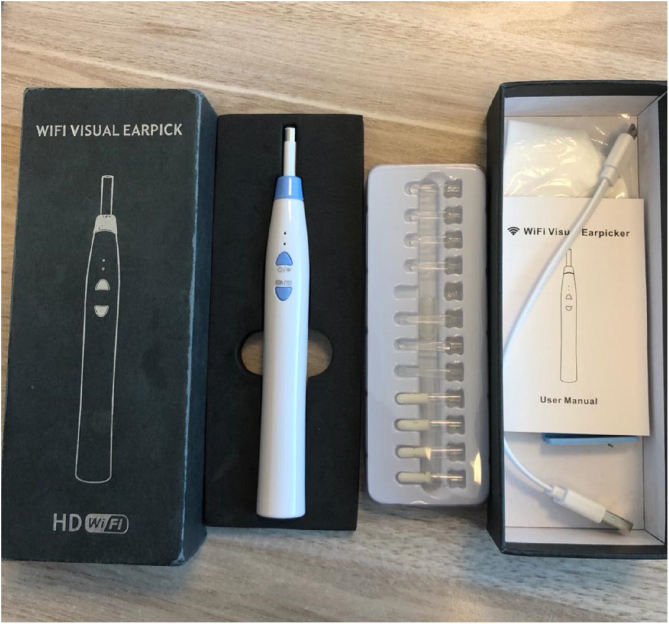
Inskam ear endoscope device.

**Figure 2:**
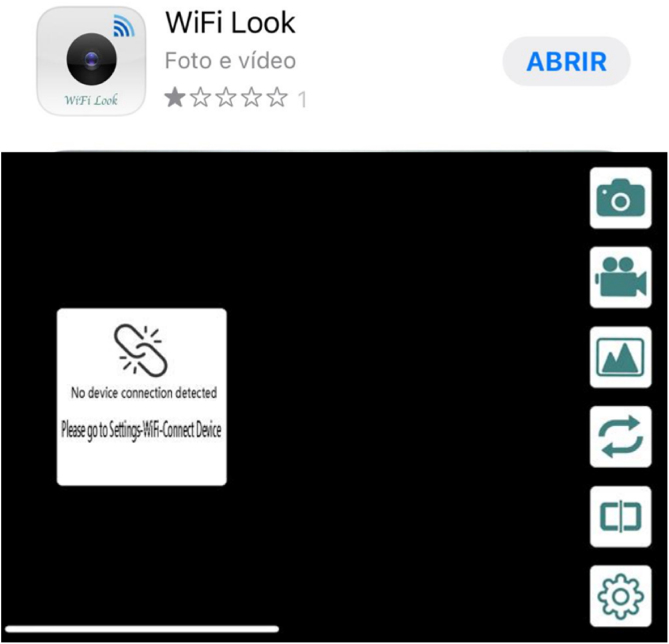
Application installed on smartphone.

The otoendoscopy was performed using a 0-degree (4 mm) Taimin rigid endoscope coupled to an HD Camera with a high resolution of 1 Megapixel and a Sony CCD Sensor with True Color technology and a Full HD coupler with a focal length – 25 mm. The smartphone device otoscopy was performed using the Inskam Ear Endoscope device (Fig. 1) via Wi-Fi connection and the application installed on the smartphone (Fig. 2).

The images obtained from otoscopies performed through the smartphone device and the rigid endoscope were recorded and stored for later analysis. The capture considered to be ideal was the one that showed sufficient image quality to allow the identification of the following anatomical parameters: integrity of the tympanic membrane boundaries (except in cases of simple chronic otitis media), presence of the entire tympanic annulus, visualization of anterior and posterior malleolar ligaments, handle of the malleus and the umbus.

At first, after obtaining the images, the recordings captured by the two devices were presented on a high-resolution monitor to an experienced otologist. The latter presented their diagnostic impression according to the viewed images and the patient's summarized clinical history. This first diagnosis was used as the gold standard for further comparison.

After this analysis, the same images (following the same presentation sequence) were displayed to a group of secondary evaluators without access to the clinical history (an experienced otorhinolaryngologist, a second-year resident in ORL, and a general practitioner). The images were evaluated regarding their perception of technical quality, their sufficiency of minimum anatomical parameters to establish a diagnosis, their possibility of defining a diagnostic hypothesis, their quality questioning based on a qualitative scale (“1 = Very Good”, “2 = Good”, “3 = Average”, “4 = Bad” and '5 = Very bad”) and its possible origin from a capture device (rigid endoscopy or smartphone otoscopy). This image assessment questionnaire can be found in Appendix 1. Image resolution quality for both the smartphone device and rigid otoendoscopy was rated related to focus and lighting in a subjective manner (i.e., whether the examiners found these parameters sufficient to assess the otoscopy).

To perform the calculation of sensitivity, specificity, positive predictive value and negative predictive value, the diagnoses provided based on the assessment of an experienced otologist (gold standard) and the assessment of the other participating professionals (generalist otorhinolaryngologist, second-year resident in ORL and general practitioner). All images obtained, regardless of their quality, were used for statistical calculation, aiming to simulate a situation more compatible with what professionals face in their daily clinical practice.

The Kappa index was used to assess the agreement between the methods, that is, rigid otoendoscopy and smartphone-enabled otoscopy. The value of *p* < 0.05 was considered statistically significant for the analysis of statistical significance. The study was submitted to the Research Ethics Committee and registered on *Plataforma Brasil*, under Opinion number 3.456.433. The study participants received the Free and Informed Consent Form.

## Results

Eighty-three otoscopies, simultaneously captured by a rigid otoendoscopy and by a smartphone-enabled otoscopy device, were included in the study, thus totaling 166 images. Five otoscopies were excluded due to impossibility of visualizing the tympanic membrane and due to obstruction of the external auditory canal by cerumen. Of these otoscopies, 20 represented normal otoscopies, 13 showed acute otitis media, 8 showed secretory otitis media, 25 with simple chronic otitis media (perforation of the tympanic membrane, only), 4 with chronic cholesteatomatous otitis media, 8 with tympanosclerosis and 5 with tympanic membrane retraction or atelectasis.

**Figure 3:**
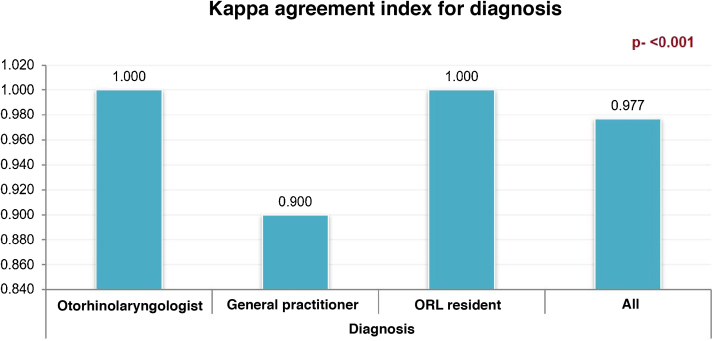
Distribution of the Kappa coefficient for evaluators in the assessment of diagnostic agreement between the smartphone device and the otoendoscopy.

There was a high rate of agreement between the diagnoses obtained from a smartphone device and rigid otoendoscopy for diagnosis, for all professionals, with a Kappa coefficient of 0.97 (*p* < 0.001). The coefficient distribution for each of the secondary evaluators is shown in Fig. 3.

When considering the mean of all evaluators in both tests, the device coupled to the smartphone showed a diagnostic sensitivity of 81.1% and a specificity of 71.1%; for the rigid otoendoscopy, the sensitivity was 84.7% and the specificity, 72.4%. The diagnostic assessment of each of the evaluators with each device used is shown in Table 1, Table 2.

**Table 1:** Diagnosis evaluation using the smartphone device.

	Sensitivity	Specificity	PPV	NPV
Otorhinolaryngologist	90.2%	83.3%	92.2%	80.2%
ORL resident	88.1%	75.0%	82.5%	72.6%
General practitioner	65.0%	55.2%	65.1%	53.4%
All	81.1%	71.1%	79.9%	68.7%

**Table 2:** Diagnosis assessment using rigid otoendoscopy.

	Sensitivity	Specificity	PPV	NPV
Otorhinolaryngologist	95.1%	85%	94%	83.1%
ORL resident	90.0%	79.0%	85.5%	77.6%
General practitioner	69.0%	53.2%	68.9%	55.1%
All	84.7%	72.4%	82.8%	71.9%

Regarding the quality of the obtained images (Table 3), the rate corresponding to “1 = Very Good” was 25.0% for otoendoscopy *versus* 13.9% for the smartphone. The classification of images as “2 = Good” was the most frequent one and it was 34.9% for the otoendoscopy *versus* 31.6% for the smartphone. The rate corresponding to “4 = Bad” was 11.7% in the otoendoscopy *versus* 20.0% in the smartphone. Finally, the rate of “5 = Very bad” was 3.9% in the otoendoscopy *versus* 4.8% in the smartphone (*p* < 0.001).

**Table 3:** Comparison of otoendoscopy and smartphone regarding image quality.

	Quality	Otoendoscopy	Smartphone	*p*
All	1 = Very good	25.0%	13.9%	
	2 = Good	34.9%	31.6%	
	3 = Average	24.4%	28.9%	<0.001
	4 = Bad	11.7%	20.8%	
	5 = Very bad	3.9%	4.8%	

**Figure 4:**
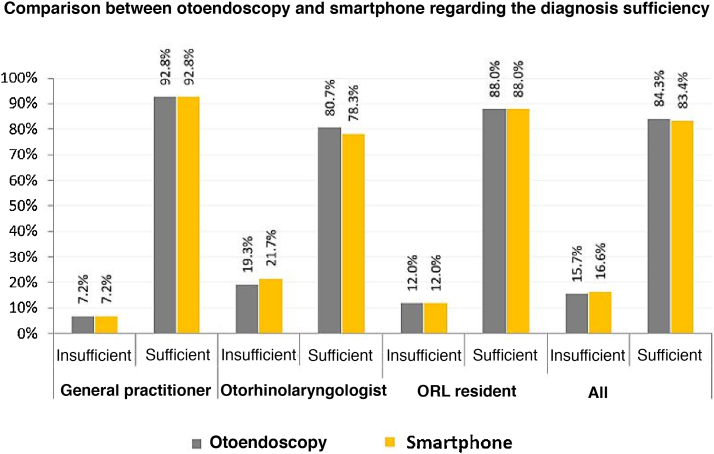
Comparison between the otoendoscopy and the smartphone regarding their sufficiency to attain the diagnosis according to each evaluator.

Regarding the sufficiency of both tests to make the diagnosis, the average of all evaluators showed that the otoendoscopy was sufficient in 84.3% and insufficient in 15%. The smartphone-enabled otoendoscopy was enough in 83.4% and insufficient in 16.6%. The classification according to each professional is shown in Fig. 4 (*p* < 0.001).

## Discussion

This study was carried out to investigate the capacity and usability of images obtained by smartphone-enabled otoendoscopy systems and to help measure their sufficiency as an instrument for professionals to make correct diagnoses of different types of middle ear pathologies, allowing the appraisal of their feasibility as a diagnostic tool in clinical practice.

An almost perfect agreement (Kappa coefficient of 0.97) was observed between the device coupled to the smartphone and the rigid otoendoscopy in attaining the diagnosis, when considering the average of all evaluators in the blinded group. A similar study carried out by Bhavana et al. demonstrated a strong correlation (Kappa coefficient of 0.76) comparing images from the rigid endoscopy with images from a smartphone without an adapted coupled device; in this case the image was captured by the smartphone with an otological speculum positioned in the patient's external ear canal.^10^

In a study by Moshtaghi et al.^11^ the smartphone-enabled otoscopic images were compared with otoscopic microscopic images and high levels of sensitivity (100%), positive predictive value (97%) and specificity (96%) were found for the smartphone device in the diagnosis of middle-ear disease. In the present study, the otoscopy performed on a smartphone device showed a diagnostic sensitivity of 81.1% and a specificity of 71.1%. Such superior results by Moshtaghi et al. can be explained by the higher image quality of the used device (consequently greater investment) in their study when compared to the present study.

Our study showed higher diagnostic sensitivity rates in the evaluation of images from the smartphone device when compared to a study that used images from a smartphone without a device coupled to it, that is, in which the image was obtained by positioning the smartphone next to an otologic speculum in the external auditory meatus.^10^ This suggests that the device can optimize image quality by adequately adapting to the ear anatomy, globally evaluating the tympanic membrane and the entire external auditory canal.

The diagnostic sensitivity of the smartphone device decreased according to the experience of the professional evaluator. Higher sensitivity and specificity rates (90.2% and 83.3% respectively) were found in the evaluation of the most experienced secondary evaluator (otorhinolaryngologist) compared to the less experienced evaluators. Similar findings were found in a study involving the comparison of micro-otoscopy images with video-otoendoscopy, reinforcing that the evaluator’s experience is also a determining factor in the diagnosis of ear pathologies, regardless of the evaluated device.^15^

The evaluators of the present study classified the images shown by the smartphone device as having good resolution, and in 83.4% of the cases they judged them to be sufficient to make the diagnosis. However, there was a decrease in the quality of some captured images and this can be explained by the difficulty in the angulation of the device's lens in patients who had external auditory meatus narrowing, directly influencing the loss of lighting and difficulties in achieving the focus, since these parameters were automatically performed by the device and depended precisely on the unobstructed positioning in the external auditory meatus.

The widespread use of smartphones by the population and also as a tool to complement medical activity is already a reality. Their use for telemedicine purposes and as resources to search for updated information has been a reality for almost a decade. More recently, its application in the field of medical diagnostics has been growing and improving the experience of in-person or remote care. However, it is a tool that needs to have its methods evaluated and, therefore, ensure not only its safety, but also its effectiveness to return satisfactory diagnoses. Thus, a device with a low investment value, with sensitivity and specificity close to that of a high-cost device such as a rigid otoendoscope, associated with the practical features of using a smartphone, may be a viable alternative in clinical routine. The applicability of this device is mainly in the diagnosis of middle and external ear diseases. In addition, the possibility of its use in remote situations, such as telemedicine, should be emphasized, and it can be used by non-specialists through adequate training, aiming at diagnostic optimization.^15^ It is extremely important to emphasize that smartphone-enabled otoscopy is a facilitating resource in the doctor–patient relationship regarding the understanding of the occurrence and clarification of one’s own disease.

The present study has some limitations that must be recognized. Only a subset of pathologies was included in the evaluators' questionnaire. Another consideration is that not all images included had ideal lighting and focus conditions, affecting the image resolution quality. However, even low-quality images were able to provide diagnostic elements that allowed helping clarify the diagnosis.

Another study limitation is that intra-examiner reliability (compared to the gold standard) was not assessed. This can be resolved through a follow-up study, using the same individuals, evaluating the same image dataset (possibly in a random order), on a subsequent date. Furthermore, this study did not assess whether the diagnostic accuracy and/or the confidence level changed as the evaluators gained more experience in reviewing digital otoscopic images, as there may be a learning curve associated with digital image-based diagnosis.

This study was based on the assumption that the reviewing otologist made the diagnoses correctly, according to the patient's clinical context. This approach can be used as the current gold standard for the diagnosis and is consistent with other studies that assessed diagnostic skills using digital imaging.^11^

New studies are required to further improve the diagnostic capacity of these devices, whether in improving the light-generating source, or in the image capture and processing system, to the possibility of using artificial intelligence that will allow the creation of reliable algorithms for a possible automated image analysis, as already present in some exams in the radiological sphere.

The use of a technological gadget adapted to a smartphone as an otoscopy image analysis tool proved to be a useful and easy-to-implement tool for different operators. The images allowed a diagnosis whose sensitivity and specificity are highly reliable in diagnosing middle and inner ear pathologies, as well as the observation of a latent and intangible improvement value regarding the perception of quality in daily medical practice.

## Conclusion

There was high diagnostic agreement between smartphone-enabled otoscopy and the rigid otoendoscopy, demonstrating the feasibility of using this device in clinical practice.

## Conflicts of interest

The authors declare no conflicts of interest.
