# Supplementary material for: Smartphone-enabled otoscopy: method evaluation in clinical practice
Source: Braz J Otorhinolaryngol. 2021 Nov 15;89(1):122–7. doi: 10.1016/j.bjorl.2021.08.012 (PMC9874339; doi:10.1016/j.bjorl.2021.08.012)
Supplement: Supplementary file 1 [file mmc1.docx]

**BJORL-D-21-00270 – Supplementary Material**

**Appendix 1** Questionnaire applied to the evaluators for each projected image.

| N. of the image: ______ | | | |
| --- | --- | --- | --- |
| 1 – Do you think this method was enough to infer the disease diagnosis through the projected image? | | | |
|  | Yes |  | No |
|  | | | |
| 2 – Does the image allow the visualization of all anatomical references of an otoscopy? | | | |
|  | Yes |  | No |
|  | | | |
| 3 – What is your diagnosis according to this image? | | | |
|  | Normal otoscopy | | |
|  | AOM | | |
|  | SOM | | |
|  | Simple COM (tympanic membrane perforation) | | |
|  | Cholesteatomatous COM | | |
|  | Tympanosclerosis | | |
|  | Tympanic membrane retraction or atelectasis | | |
|  | | | |
| 4 – On a scale of 1 to 5, how would you rate the quality of the resolution of this image (considering lighting and focus)? | | | |
|  | 1 – Very good | | |
|  | 2 – Good | | |
|  | 3 – Average | | |
|  | 4 – Bad | | |
|  | 5 – Very bad | | |
|  | | | |
| 5 – Do you suppose this image comes from a smartphone or rigid endoscopy? | | | |
|  | Smartphone | | |
|  | Otoendoscopy | | |
